# Mental Health Professionals’ Perceptions of Benefits and Disadvantages of Telehealth: International Mixed Methods Study

**DOI:** 10.2196/75905

**Published:** 2026-03-04

**Authors:** Madeline I Montoya, Cary S Kogan, Brigitte Khoury, José A García-Pacheco, Rebeca Robles, Tahilia J Rebello, Maya Kulygina, Geoffrey M Reed

**Affiliations:** 1 School of Psychology University of Ottawa Ottawa, ON Canada; 2 Department of Psychiatry American University of Beirut Medical Center Beirut Lebanon; 3 Centro de Investigación y Docencia Económicas Mexico City Mexico; 4 Center for Global Mental Health Research Instituto Nacional de Psiquiatría Ramón de la Fuente Muñiz Mexico City Mexico; 5 Columbia University Vagelos College of Physicians and Surgeons New York, NY United States; 6 Training and Research Centre, Mental-Health Clinic No.1 Named After N.A. Alekseev Moscow Russian Federation; 7 Department of Psychiatry Columbia University Vagelos College of Physicians and Surgeons New York, NY United States

**Keywords:** global mental health, telehealth, mental health professionals, COVID-19, implementation science

## Abstract

**Background:**

Telehealth has become an integral component of mental health care delivery worldwide. Understanding provider perceptions is essential to guiding its continued implementation.

**Objective:**

This international study used quantitative and qualitative methodologies to examine and broaden our understanding of the benefits and concerns related to telehealth for mental health care.

**Methods:**

An internet-based survey was conducted during the COVID-19 pandemic between November 11 and December 18, 2020, among mental health professionals, primarily psychiatrists and psychologists, registered with the World Health Organization’s Global Clinical Practice Network. Clinicians completed the survey in 1 of 6 languages (Chinese, English, French, Japanese, Russian, or Spanish). Descriptive statistics and logistic regressions were used to analyze quantitative survey data on concerns and implementation of telehealth. Responses to an open-ended question about providers’ perspectives on the benefits of telehealth were analyzed qualitatively.

**Results:**

In total, 847 participants completed the telehealth section of the survey, and 496 provided a response to the open-ended question. Quantitative data on telehealth use and concerns revealed that clinicians’ primary concerns focused on technical issues and clinical effectiveness relative to in-person services, specifically, loss of clinical information (eg, nonverbal behavior) and challenges with establishing a therapeutic alliance. Findings varied by profession, World Health Organization region, and telehealth training and experience. Qualitative data examining benefits fell into 3 major areas: accessibility and reach of mental health services, efficiency and flexibility for clinicians, and enhancement of clinical processes and outcomes. Taken together, findings revealed a trade-off between telehealth benefits and disadvantages.

**Conclusions:**

From the perspective of mental health professionals, telehealth practice comes with key challenges and valuable benefits. Findings offer important considerations for the implementation of telehealth systems, including the importance of training and education and balancing trade-offs to optimize care.

## Introduction

Following the onset of the COVID-19 pandemic and subsequent disruption of mental health services, clinicians and health care systems around the world experienced an abrupt shift to remote delivery of mental health services [1,2]. The term telehealth will be used, which is defined by the World Health Organization (WHO) as “the delivery of health care services, where patients and providers are separated by distance” [3]. Telehealth encompasses the use of information communication technology for the diagnosis and treatment of diseases and injuries, research, and continuing education of health professionals. This broad definition will include services provided by all health professionals working in mental health (eg, psychiatry and psychology) across various modalities, both synchronous (eg, videoconferencing or telephone) and asynchronous (eg, SMS text message or email).

Telehealth became central for continuity of mental health care throughout the pandemic and as a solution to meeting increased demand [4,5]. Governments and systems launched digital platforms [6,7] and eased regulatory, policy, and billing restrictions [8], while professional bodies produced guidelines to assist clinicians [9,10]. These combined efforts led to a substantial global surge in telehealth use for mental health care, which has remained robust in the postpandemic era [11,12].

Numerous studies conducted prior to and during the COVID-19 pandemic have shown favorable perceptions about telehealth including high client satisfaction and provider acceptability [1,13]. The evidence has demonstrated the feasibility and effectiveness of telehealth compared to in-person services for the assessment and treatment of various mental disorders and patient populations [14-16]. Telehealth can improve access to care in underserved areas and for individuals who typically experience barriers to accessing in-person services [1]. Telehealth can also be convenient (eg, reducing burdensome travel [17], cost-effective, and time-efficient for patients and providers [18]). Telepsychiatry research suggests additional advantages such as reduced psychiatric admissions [19,20] and greater scheduling flexibility [21]. However, the literature on the benefits of telehealth is fragmented, lacking a cohesive perspective across mental health providers and geographic regions.

Despite largely positive accounts of the potential of telehealth, numerous challenges still exist. Research conducted prior to and during the COVID-19 pandemic found that mental health professionals from various countries remain concerned with clinical elements of telehealth such as quality or effectiveness of care compared to in-person services [2,18] and assessing patients remotely. Technological challenges remain a barrier to telehealth use, particularly technical issues (eg, poor connectivity) and client access to technology [1,22,23]. Finally, although professional guidelines and easing of restrictions addressed many ethical, regulatory, and legal barriers to telehealth, some studies show that providers during the pandemic still reported concerns about patient privacy and confidentiality [2]. Therefore, it is important to comprehensively examine which clinical, technological, and ethical concerns are most challenging for mental health providers. For example, the specific sources that contribute to providers’ perceptions of telehealth as less effective relative to in-person services remain unclear. Several studies suggest that mental health professionals report difficulty establishing a therapeutic alliance with their clients via telehealth [19,24], although findings are mixed [25]. Others report difficulty with interpreting patients’ nonverbal communication [26]. Still other research suggests that difficulties with therapeutic skills (eg, showing empathy) is also a factor [27].

The prevailing view is that telehealth is a largely effective and more equitable means of service provision that is expected to remain a significant component of service provision. However, it may not be appropriate or effective for everyone. More evidence is needed to understand if telehealth is feasible and effective for certain patient populations and types of services [1,15]. Therefore, mental health professionals must assess the advantages and disadvantages of offering telehealth services in their particular contexts with specific patients. Moreover, given that clinicians are considered gatekeepers to implementation and sustainability of telehealth for mental health care [23], understanding provider perceptions is key to informing continued use.

The COVID-19 pandemic created a unique opportunity to assess the perceptions of telehealth providers not only from numerous clinical settings and geographical locations but also from professionals with greater generalizability than the subset of participants in prepandemic telehealth studies who may have been influenced by selection bias (ie, interest in telehealth) and existing infrastructure for virtual care. Therefore, research conducted during the pandemic contains informative perspectives from both groups of mental health providers: those forced to transition to telehealth and those familiar with and already implementing telehealth.

This mixed methods study used distinct but complementary qualitative and quantitative research questions to obtain more comprehensive information on the experiences, concerns, and benefits of telehealth from the perspective of international mental health providers during the COVID-19 pandemic [28,29]. Participants were drawn from the WHO’s Global Clinical Practice Network (GCPN), a multilingual and international network of psychologists, psychiatrists, and other mental health professionals from 160 countries representing all WHO global regions [30]. It was hypothesized that the perspectives of mental health professionals would mirror previous findings on the advantages and disadvantages of telehealth as well as illustrate differential experiences across contexts (eg, world region) and professional demographics (eg, discipline, telehealth training, and experience [1,2]). Telehealth research often examines psychologists and psychiatrists either separately (ie, telepsychiatry) or treats them as a single group despite meaningful differences in the scope of practice (eg, medication management and physical health assessments). This study examines differences between the 2 professions, as discipline was hypothesized to shape telehealth use and perceptions. The term clinician is used to refer collectively to psychologists, psychiatrists, and other mental health professionals registered with the GCPN.

## Methods

### Study Design, Participants, and Procedure

This study was part of the “Longitudinal COVID-19 Survey of Mental Health Professionals Project,” a 3-wave longitudinal internet-based survey study examining the effects of the COVID-19 pandemic on global mental health professionals’ practice including telehealth [2] and occupational well-being [31]. This study focuses on the survey questions related to telehealth included in the second wave of data collection, which occurred between November 11 and December 18, 2020 (for a total data collection period of 5 weeks). A convergent mixed methods design was used, in which quantitative and qualitative data were collected in parallel (ie, in the same survey), analyzed separately with equal priority, and then integrated using a joint display of the 2 forms of data to provide additional insights [32]. This study used the GRAMMS (Good Reporting of a Mixed Methods Study) criteria [33] to guide reporting (Multimedia Appendix 1).

The Longitudinal COVID-19 Survey of Mental Health Professionals Project procedures and recruitment for the telehealth section of the survey have been described elsewhere in more detail [2]. In brief, participants were recruited from the GCPN. To be eligible for registration with the GCPN, providers must have completed their training and be qualified to provide mental health services in their countries. Initially, GCPN members registered because of interest in participating in field studies related to the *International Classification of Diseases, 11th Revision* (*ICD-11*) being conducted by the WHO [30].

The online survey was administered using Qualtrics in Chinese, English, French, Japanese, Russian, and Spanish. Content was initially developed in English and then translated by fluent experts affiliated with the GCPN’s International Advisory Group.

GCPN members proficient in the corresponding survey languages were invited to participate via email containing an individualized survey. Upon accessing the link, participants were presented with a description of the study. Reminder emails were sent 7 and 14 days after the initial email. Participants who indicated that they had provided direct clinical services in the past 2 weeks and had used telehealth for either new or continuing patients were directed to the telehealth survey questions.

### Ethical Considerations

The study was approved by the institutional review board at Columbia University/New York State Psychiatric Institute (registration: #6886) and the University of Ottawa (registration: H-06-20-5973). Participants, who were practicing mental health professionals and members of the GCPN, were asked to provide consent for participation by selecting “Yes” to proceed with the online survey after reviewing a description of the study. Participants were not compensated for participation. Study data were deidentified to maintain participant privacy and confidentiality.

### Survey

Quantitative data were collected from closed-ended items about telehealth concerns and implementation, and qualitative data were collected from an open-ended question about clinicians’ perspectives on the benefits of telehealth. Telehealth survey items were developed from a review of the existing literature at the time (including data from the first-wave telehealth survey published in Montoya et al [2]), together with group discussion and expert review by the authors and the GCPN’s International Advisory Group (Multimedia Appendix 2).

Upon starting the survey, participants were informed that they would be asked about their recent work activities and to have their work calendars accessible when completing the survey. Participants were about years of telehealth experience, if they had received training on technological, ethical, legal, and clinical aspects of telehealth service delivery or no training at all, and current (ie, past 2 weeks) telehealth use. Demographic and telehealth use survey items included closed-ended and multiple response (or check-all-that-apply) questions. Likert-type questions about perceived effectiveness of telehealth for specific age groups and treatment modality (eg, individuals and couples) were also included.

To identify specific challenges, 4 separate checklists were presented, which focused on clinical, technical, ethical, legal, and professional, and finally, administrative elements of providing telehealth services. If participants endorsed concerns about “clinical effectiveness relative to in-person services” in the checklist of clinical elements, they were directed to an additional checklist containing more detailed concerns related to clinical effectiveness (eg, building and maintaining a therapeutic alliance and showing empathy). These options for specific concerns were based on the telehealth literature [13] as well as basic clinical skills that are considered important for therapeutic change [34,35]. Participants could select multiple items for each checklist and use an open-text field to specify an “other” response.

The following open-ended question was included to capture participants’ perspectives on the benefits of telehealth: “In addition to making it possible to consult with patients remotely, have there been any benefits of using telehealth services?”

### Quantitative Data Analysis

Of a total of 15,179 GCPN members who were invited to participate in the larger study, 2124 (13.9%) agreed to participate in the survey, and 2088 (98.3%) of those participants provided sufficient data for analysis (ie, <90% missing survey data). Of those participants, 847 completed the telehealth section. Descriptive and chi-square statistics were used to summarize and compare quantitative survey data. Logistic regressions were used to examine predictors of clinicians’ common concerns. Statistical calculations were performed using SPSS (version 29; IBM Corp).

### Qualitative Data Analysis

The purpose of the qualitative data analysis was to examine and summarize the content of clinicians’ responses. The analysis was guided by recommendations from Miles et al [29] and consisted of first- and second-cycle coding methods. First cycle coding is a way of assigning initial descriptive codes to the data, whereas second cycle coding is the reorganization of these first cycle codes into meaningful themes, categories, or concepts [36].

Of the 847 clinicians who completed the telehealth section survey, 496 (58.6%) provided free-text responses to the open-ended question. Responses ranged from 1-word phrases to a paragraph; however, most were a few sentences in length. In general, characteristics of those who completed the open-ended question were similar to those who did not. Significant differences were found for telehealth training, with a greater proportion of participants with telehealth training answering the open-ended question (*χ*^2^_1_=12.1; *P*<.001). Moreover, those who responded to the open-ended question were older (t_845_=4.6; *P*<.001) and had slightly more years of clinical experience (t_845_=2.8; *P*=.003). All 496 responses were imported to and analyzed using NVivo (version 14; Lumivero). Two of the authors (MIM and CSK) coded the data and consulted with a researcher with experience in approaches relevant to this study. Coders were either actively or passively fluent in the 3 most common survey languages (English, Spanish, and French); thus, 421 (84.5%) responses were kept in their original language until the end of data analysis. MIM, the primary coder, was fluent in the former 3 languages. CSK was fluent in English and French and could read and understand Spanish. Chinese, Japanese, and Russian responses were translated to English prior to coding using DeepL Translator, a machine translation tool known for its accuracy and natural, high-quality translations. DeepL Translator is owned by DeepL SE, a German-based company that uses artificial intelligence to offer various translation supports.

Following an inductive approach, MIM and CSK used initial coding (an open-ended, data-driven coding process [36]) and in vivo coding (a coding technique that uses participants’ exact words or phrases to extract ideas [36]) to independently code 150 responses (33% of the data). This initial sample was selected by sorting the full sample of responses by WHO region and coding a proportional number of responses based on WHO region and language. Responses were intentionally skipped as needed to ensure proportional representation. For example, 10% (15/150) of responses were from Western Pacific-Asia; thus, 15 responses from this region were coded as part of the initial sample of 150 responses. Next, coders shared their initial codes to collaboratively develop a preliminary codebook.

Discrepancies in code definitions, labels, and interpretations were resolved through discussion until consensus was reached. Using this initial codebook, a process of intercoder reliability was conducted. MIM and CSK independently coded a sample of 100 (20%) responses different from the initial sample that was used to develop initial codes. κ statistics (overall κ=0.65) and percentage agreement (ranging from 65% to 100%) were used to identify codes with lower agreement. The coders discussed inconsistencies until consensus was reached. Codes were merged, dropped, and refined until a final codebook was generated. MIM then coded all 496 responses.

Two second-cycle coding methods were used: pattern and axial coding. MIM conducted a process of pattern coding to identify emergent themes, configurations, or explanations in the data [29]. Axial coding was used to distinguish properties and dimensions of emergent categories [36] such as benefits specific to patients versus clinicians. Experiential differences and similarities across the WHO region and profession were also examined. MIM and CSK discussed and developed emergent themes and overall conceptualization. The relative frequency with which codes were applied was used to distinguish between topics that arose more often than others to better characterize the diversity of perceptions reported by clinicians [37].

Efforts were taken to ensure the quality of the data analysis. Methodological and analytic documentation (eg, memo writing) was used to keep track of the coding process and related decisions [36]. During meetings, MIM and CSK attempted to maintain reflexivity and note sources of bias during analysis (eg, the impact of one’s own sociocultural background on data interpretation). Furthermore, coders were blinded to all demographic information when analyzing codable text except for language and profession. The rationale was that access to this information could help contextualize responses (eg, assessment could include physical examination in psychiatry). Coders searched for disconfirming evidence (ie, counterexamples to recurring patterns), and that no substantively new themes or patterns emerged in the final stages of coding the data sample [29].

### Integrating Quantitative and Qualitative Data

After separate analyses, frequently endorsed concerns (quantitative data) and identified benefits (qualitative data) were brought together in a table for elaboration [32]. MIM and CSK met to conduct a higher-level interpretation of findings to supplement and enhance understanding of the advantages and disadvantages of telehealth for mental health care [28].

## Results

### Quantitative: Telehealth Use and Concerns

#### Overview

Clinician demographic and professional characteristics are provided in Table 1. Participants represented all WHO regions and 73 countries. Most worked in mental health settings (754/847, 89%), followed by substance abuse specialty programs (200/847, 23.6%), university or other educational institutions (182/847, 21.5%), other general medical settings (148/847, 17.5%), primary care settings (67/847, 7.9%), and finally, hospital emergency departments (15/847, 1.8%). Approximately half of the participants (446/847, 52.7%) reported working in multiple settings. Most participants (776/847, 91.6%) reported that they had provided mental health services (such as diagnostic assessment, psychological assessment, or psychotherapy) during the past 2 weeks. Additionally, 84.8% (263/310) of psychiatrists had also provided psychopharmacotherapy, and 29% (90/310) had provided other medical services (including general medical services and management of comorbid medical conditions).

Participants reported using various modalities for direct clinical services (assessment, treatment, or monitoring of patients) including in person (607/847, 71.7%), videoconferencing (632/847, 74.6%), telephone (654/847, 77.2%), chat (353/847, 41.7%), and email (359/847, 42.4%). Psychiatrists were more likely to see patients in person compared to psychologists (*χ*^2^_1_=28.5; *P*<.001). Table 2 provides the number of hours spent and patients seen using telehealth for psychiatrists and psychologists in the past 2 weeks.

Table 3 shows providers’ perceived effectiveness of telehealth by treatment modality (individual, group, families, and couples) and patient age groups (older adults, adults, adolescents, and children).

**Table 1:** Clinician demographics and occupational characteristics.

		Sample of telehealth participants (N=847)
**Gender, n (%)**		
	Men	372 (43.9)
	Women	472 (55.7)
	Other self-identified	3 (0.4)
**Language, n (%)**		
	English	454 (53.6)
	Spanish	174 (20.5)
	Japanese	67 (7.9)
	Chinese	29 (3.4)
	French	53 (6.3)
	Russian	70 (8.3)
**WHO^a^ region^b^, n (%)**		
	Africa	22 (2.6)
	Americas-South	135 (15.9)
	Americas-North	156 (18.4)
	Eastern Mediterranean	13 (1.5)
	Europe	343 (40.5)
	South-East Asia	48 (5.7)
	Western Pacific-Asia	106 (12.5)
	Western Pacific-Oceania	24 (2.8)
**Country income level, n (%)**		
	Low	4 (0.5)
	Lower-middle	87 (10.3)
	Upper-middle	219 (25.9)
	High	537 (63.4)
**Profession, n (%)**		
	Psychiatry	310 (36.6)
	Psychology	384 (45.3)
	Other^c^	153 (18.1)
**Age (years)**		
	Mean (SD)	52.3 (11.3)
	Range	25-89
	Median (IQR)	52 (43-60)
**Years of experience**		
	Mean (SD)	20.9 (10.2)
	Range	0-57
	Median (IQR)	20 (13-29)
**Telehealth training, n (%)**		
	Yes	475 (56.1)
	No training	372 (43.9)
**Telehealth experience, n (%)**		
	Less than 1 year	419 (49.5)
	More than 1 year	428 (50.5)

^a^WHO: World Health Organization.

^b^Participants were predominantly from the following countries in each WHO region—Africa: Nigeria, South Africa; Americas-South: Mexico, Argentina, Brazil, Columbia, Peru; Americas-North: the United States, Canada; Eastern Mediterranean: United Arab Emirates, Egypt; Europe: Spain, Russia, Germany, the United Kingdom, France, Denmark, Switzerland; South-East Asia; India; Western Pacific-Asia: Japan, China; and Western Pacific-Oceania: Australia, New Zealand.

^c^Other professionals included predominantly social work, nursing, counseling, occupational therapy, and sex therapy.

**Table 2:** Number of patients seen and hours spent using telehealth in the past 2 weeks.

		Values, n	Psychologist, mean (SD)	Values, n	Psychiatrist, mean (SD)
**Telephone**		263		245	
	Patients		7.79 (13.34)		8.48 (9.27)
	Hours		7.40^a^ (10.06)		5.79^a^ (7.09)
**Videoconferencing**		332		201	
	Patients		10.97 (13.36)		9.83 (11.77)
	Hours		14.96^a^ (17.12)		10.66^a^ (12.29)
**Instant messaging**		126		141	
	Patients		5.82 (10.08)		7.77 (10.26)
	Hours		6.53 (14.41)		5.35 (10.04)
**Email**		137		114	
	Patients		3.58 (5.21)		4.81 (4.88)
	Hours		2.52 (3.59)		2.31 (2.76)

^a^Significantly different at *P*<.05.

**Table 3:** Clinicians who reported that the telehealth services they provided for specific patient populations were about the same or more effective than in-person by treatment modality and patient age groups.

		Value, n/N (%)
**Patient age groups**		
	Adults (65+ years)	155/406 (38.2)
	Adults (19-64 years)	468/751 (62.3)
	Adolescents (13-18 years)	192/359 (53.5)
	Children (0-12 years)	42/188 (22.3)
**Treatment modality**		
	Groups	68/200 (34)
	Families	88/271 (32.5)
	Couples	106/270 (39.3)
	Individual assessment and treatment	425/774 (54.9)

Table 4 lists the most common concerns (endorsed by 40% or more) regarding clinical, technical, ethical, legal, professional, and administrative aspects of telehealth provision. Psychologists were significantly more concerned about a lack of private space (*χ*^2^_1_=7.6; *P*=.006) and technical issues (*χ*^2^_1_=4.8; *P*=.03) when compared to psychiatrists. Conversely, psychiatrists were significantly more concerned with remote assessment of patients (*χ*^2^_1_=32.5; *P*<.001), assessing high-risk patients and managing emergencies (*χ*^2^_1_=12.4; *P*<.001), showing empathy (*χ*^2^_1_=5.6; *P*=.02), and licensing or credentialing requirements (*χ*^2^_1_=36.1; *P*<.001) when compared to psychologists.

**Table 4:** List of most frequent telehealth concerns endorsed by at least 40% of psychiatrists, psychologists, and/or other professionals (mainly social work, nursing, counseling, occupational therapy, and sex therapy).

				Psychiatrist, n (%)		Psychologist, n (%)		Other, n (%)		Total, n (%)
**Clinical concerns**										
	**Clinical effectiveness relative to in-person services**		196 (68.5)		224 (60.7)		86 (57)		506 (62.8)	
		Loss of clinical information	158 (81.4)		175 (78.8)		63 (75)		396 (79.2)	
		Building and maintaining a therapeutic alliance	141 (72.7)		150 (67.6)		60 (71.4)		351 (70.2)	
		Showing empathy	103 (53.1)		92 (41.4)		42 (50)		237 (47.4)	
		Implementing therapeutic interventions	68 (35.1)		97 (43.7)		43 (51.2)		208 (41.6)	
	Assessment of high-risk patients and managing emergencies remotely		194 (67.8)		189 (51.2)		77 (51)		460 (57.1)	
	Lack of private space for patients during telehealth sessions		128 (44.8)		199 (53.9)		60 (39.7)		387 (48)	
	Remote assessment of patients (eg, diagnostic assessments, self-report measures, lack of opportunity for physical examinations, and medication blood level monitoring)		171 (59.8)		129 (35)		58 (38.4)		358 (44.4)	
	Decrease in patient engagement compared to in-person services		119 (41.6)		151 (40.9)		70 (46.4)		340 (42.2)	
**Technological concerns**										
	Technical issues (eg, audio or video quality and stability of internet connection)		211 (71)		294 (78.4)		116 (78.9)		621 (75.8)	
	Patients’ access to equipment		177 (59.6)		230 (61.3)		93 (63.3)		500 (61.1)	
	Patient familiarity with connecting		137 (46.1)		166 (44.3)		77 (52.4)		380 (46.4)	
**Ethical, legal, and professional concerns**										
	Patient consent, privacy, security, and confidentiality		112 (42.6)		128 (38.6)		61 (42.4)		301 (40.7)	
	Licensure or credentialing requirements		116 (44.1)		70 (21.1)		34 (23.6)		220 (29.8)	
**Administrative concerns**										
	Disruption of routine and workflow		121 (40.7)		131 (34.9)		58 (39.5)		310 (37.9)	

#### Logistic Regressions

When chi-square tests showed associations between at least 2 demographic variables and the most common concerns, binomial logistic regressions were used to determine whether demographic and professional variables affected the likelihood that clinicians endorsed a common concern. Predictors included age, years of clinical experience, profession (psychiatrists, psychologists, and other professionals), telehealth experience (<1 year vs >1 year), telehealth training received (no vs yes), and WHO region. Age and years of clinical experience did not significantly contribute to overall model fit and were thus excluded from the final models (Table 5).

**Table 5:** Logistic regression of predictors of common telehealth concerns.

Predictors				OR^a^ (95% CI)	*P* value
**Clinical effectiveness relative to in-person services**					
	**Profession^b^**				
		Psychiatrist	1.336 (0.847-2.106)		.21
		Psychologist	1.045 (0.697-1.566)		.83
	**WHO^c^ region^d^**				
		Africa	1.671 (0.631-4.428)		.30
		Americas-South	1.301 (0.778-2.175)		.32
		Eastern Mediterranean	3.691 (0.771-17.662)		.10
		Europe	1.314 (0.853-2.024)		.22
		South-East Asia	1.394 (0.695-2.795)		.35
		Western Pacific-Asia	1.408 (0.779-2.545)		.26
		Western Pacific-Oceania	1.179 (0.482-2.886)		.72
	Telehealth training^e^ (none)		1.378 (1.011-1.878)		.04
	Telehealth experience^f^ (<1 year)		1.348 (1.004-1.812)		.047
**Remote assessment of patients**					
	**Profession^b^**				
		Psychiatrist	2.079 (1.319-3.276)		.002
		Psychologist	0.901 (0.595-1.363)		.62
	**WHO region^d^**				
		Africa	2.077 (0.791-5.453)		.14
		Americas-South	0.920 (0.545-1.551)		.75
		Eastern Mediterranean	1.167 (0.354-3.855)		.80
		Europe	0.753 (0.482-1.175)		.21
		South-East Asia	2.047 (1.016-4.124)		.045
		Western Pacific-Asia	1.877 (1.036-3.401)		.04
		Western Pacific-Oceania	0.720 (0.284-1.823)		.49
	Telehealth training^e^ (none)		1.429 (1.050-1.944)		.02
	Telehealth experience^f^ (<1 year)		1.010 (0.751-1.358)		.95
**Lack of private space for patients during telehealth sessions**					
	**Profession^b^**				
		Psychiatrist	1.447 (0.922-2.271)		.11
		Psychologist	1.837 (1.224-2.759)		.003
	**WHO region^d^**				
		Africa	0.758 (0.301-1.908)		.56
		Americas-South	1.372 (0.822-2.290)		.23
		Eastern Mediterranean	0.500 (0.152-1.641)		.25
		Europe	0.658 (0.428-1.012)		.06
		South-East Asia	1.048 (0.532-2.064)		.89
		Western Pacific-Asia	0.521 (0.292-0.930)		.03
		Western Pacific-Oceania	0.819 (0.337-1.990)		.66
	Telehealth training^e^ (none)		1.242 (0.920-1.677)		.16
	Telehealth experience^f^ (<1 year)		1.043 (0.783-1.389)		.77
**Technical difficulties**					
	**Profession^b^**				
		Psychiatrist	0.923 (0.547-1.558)		.77
		Psychologist	1.042 (0.635-1.707)		.87
	**WHO region^d^**				
		Africa	0.739 (0.242-2.262)		.60
		Americas-South	0.875 (0.461-1.659)		.68
		Eastern Mediterranean	0.423 (0.115-1.564)		.20
		Europe	0.695 (0.403-1.197)		.19
		South-East Asia	0.674 (0.294-1.545)		.35
		Western Pacific-Asia	0.362 (0.189-0.696)		.002
		Western Pacific-Oceania	0.478 (0.181-1.265)		.14
	Telehealth training^e^ (none)		0.920 (0.653-1.297)		.64
	Telehealth experience^f^ (<1 year)		1.556 (1.115-2.173)		.009
**Patient consent, privacy, security, and confidentiality**					
	**Profession**				
		Psychiatrist	0.720 (0.399-1.301)		.28
		Psychologist	0.742 (0.430-1.282)		.29
	**WHO region**				
		Africa	1.504 (0.446-5.069)		.51
		Americas-South	0.199 (0.095-0.415)		<.001
		Eastern Mediterranean	0.926 (0.240-3.577)		.91
		Europe	0.700 (0.400-1.226)		.21
		South-East Asia	3.055 (1.095-8.521)		.03
		Western Pacific-Asia	0.681 (0.327-1.417)		.30
		Western Pacific-Oceania	0.535 (0.165-1.739)		.30
	Telehealth training (none)		0.802 (0.548-1.175)		.26
	Telehealth experience (<1 year)		0.745 (0.511-1.085)		.13

^a^OR: odds ratio.

^b^Other professionals as reference.

^c^WHO: World Health Organization.

^d^Americas-North as reference.

^e^Received telehealth training as reference.

^f^More than 1 year of telehealth experience as reference.

For clinical concerns of telehealth, the model investigating endorsement of clinical effectiveness relative to in-person services was statistically significant (*χ*^2^_11_=21.4; Nagelkerke *R*^2^=0.04; *P*=.03). A lack of telehealth training and less than 1 year of telehealth experience were significant predictors of the likelihood of clinicians reporting clinical effectiveness of telehealth as a concern. The model predicting concern with remote assessment of patients was statistically significant (*χ*^2^_11_=71.0; Nagelkerke *R*^2^=0.14; *P*<.001). The model suggested that psychiatrists, clinicians from South-East Asia and Western-Pacific Asia (compared to clinicians from Americas-North), and clinicians with no telehealth training were more likely to report remote assessment as a telehealth concern. The model predicting concern with lack of private space for patients was also statistically significant (*χ*^2^_11_=30.0; Nagelkerke *R*^2^=0.05; *P*=.002) with psychologists, clinicians from Americas-North (compared to clinicians from Western Pacific-Asia) being more likely to endorse this concern.

Regarding technological concerns, the model predicting endorsement of technical difficulties (eg, audio or video quality and stability of internet connection) was statistically significant (*χ*^2^_11_=30.0; Nagelkerke *R*^2^=0.05; *P*=.007). Clinicians with less than 1 year of telehealth experience were more likely to report technical difficulty as a concern. Conversely, compared to participants living in the Americas-North, clinicians in Western-Pacific Asia were less likely to endorse technical difficulties. Finally, the model predicting concern with patient consent, privacy, security, and confidentiality was statistically significant (*χ*^2^_11_=48.5; Nagelkerke *R*^2^=0.12; *P*<.001). The WHO region was the only significant predictor. Compared to clinicians living in Americas-North, clinicians in Americas-South were less likely to endorse concern with patient consent, privacy, security, and confidentiality, whereas clinicians in South-East Asia were conversely more likely to report this concern.

### Qualitative Results: Benefits of Telehealth

#### Overview

Three key themes related to the benefits of telehealth were identified: accessibility and reach of mental health services, efficiency and flexibility for clinicians, and enhancement of clinical process and outcomes. In general, clinicians’ responses highlighted multiple benefits of telehealth. Codes related to flexibility and convenience, increased access, and time savings were most commonly and consistently cited across the WHO region and profession. However, the context and conditions in which respondents described these codes differed and thus gave rise to several subthemes.

Themes and their respective subthemes are presented in the section below, with representative quotes. Representative responses written in any of the 5 other languages other than English are shown in their original form alongside an English translation. Where descriptive differences by WHO region or profession were observed, these are discussed within corresponding themes. Salient or evocative codes that did not fit the main themes are discussed further below.

#### Theme 1: Accessibility and Reach of Mental Health Services

This theme was developed primarily from a review of responses where codes related to flexibility and convenience for patients, time savings, and greater access to services were applied. The following 3 subthemes were included:

#### Reduces Barriers to Care

Clinician responses suggested that telehealth reduces burdens and barriers associated with accessing and receiving in-person mental health services. Many providers indicated that telehealth improved access by reducing geographical and logistical barriers. For example: “Reaching easily patients that live far or in islands where there are no mental health services” (Psychiatrist, Greece).

Others described a reduction in costs and stress that come with traveling to in-person appointments for patients: “Convenience for families not needing to drive/find parking/childcare for siblings etc.” (Psychologist, Australia).

Several clinicians described how telehealth increased access for specific populations that typically experience challenges with attending in-person services including older people, individuals with physical and mental comorbidities, and those with mobility difficulties: “It has meant much greater access for people with disabilities and people with more full schedules, which is fantastic” (Psychologist, the United States).

#### Expands Reach and Cost-Effectiveness of Mental Health Service Delivery

Many clinicians described being able to contribute to the creation of greater access through telehealth. This was indicated on both a provider and a systemic level. For instance: “It has meant I can work with people throughout the state(s) I am licensed in, which creates much more access” (Psychologist, the United States).

Some clinicians reported being able to “see” more patients and having “mas cobertura” (“greater coverage”). Whereas others, such as a psychiatrist in India, cited greater “availability of services in distress, emergency and follow up.”

Some also indicated a reduction in overhead expenses. For example, a psychologist in China reported feeling “... less financially burdened for office space.” Similarly, a psychologist in Mexico reported, “disminución en costos de alquiler de consultorio” (“Decrease in office rental costs”).

#### Safety of Services Provided

This subtheme came from a code specific to the context of the COVID-19 pandemic, which focused on reduced risk to health. For instance, a psychiatrist in India noted that telehealth, “... reduces risk of exposure to COVID-19 both for the doctor and patient and his/her family.”

#### Theme 2: Efficiency and Flexibility for Clinicians

This theme was developed from codes related to flexibility and convenience for mental health professionals, time savings, provider self-care, and access to other professionals. A review of these texts illustrated the benefits of telehealth that were provider-specific in either a personal or professional capacity. The following 2 subthemes emerged:

#### Enables Clinicians to Use Their Work Time More Effectively and Flexibly

Many clinicians discussed better time management as a benefit of telehealth. For example, a psychiatrist in Switzerland indicated: “Organisation plus facile de l’agenda.” (“Easier to manage my schedule.”). Moreover, clinicians described how this flexibility allowed them more time to pursue personal, domestic, or professional activities:

He podido aprovechar mejor los espacios intermedios, prácticamente no tengo horarios muertos. (I have been able to take better advantage of the time between appointments. I have practically no dead time.)

Psychologist, Mexico

I can work from home and be available to my partner in a more immediate way, and I can utilize the resources in home more flexibly. I save 6-8 hours a week in commuting time.

Psychologist, the United States

Seguir trabajando en otros ámbitos. (Continue working in other areas.)

Psychologist, Argentina

#### Promotes Provider’s Well-Being and Access to Other Professionals

Some providers reported that working from home promoted their capacity for self-care and well-being. For example:

[M]oins d’énergie dépensée en préparatifs et déplacements = moins fatigué. ([L]ess energy spent on preparation and travel = less tired.)

Psychologist, Canada

Me siento más relajada al poder acceder fácilmente a tomar algún alimento o descansar entre consulta y consulta en un espacio diferente, otro sillón, la cama, el jardín, etc. (I feel more relaxed as I can easily get something to eat or rest between sessions in a different space, another couch, the bed, the garden etc.)

Psychologist, Mexico

Some clinicians also reported easier access to professional development opportunities (eg, “docencias (teachings)” and “capacitaciones (trainings)”), colleagues, and clinical meetings:

Attending multiagency meetings, which are sometimes many miles from the previous appointment, are now attendable via MS Teams, just a couple of clicks away.

Psychiatrist, the United Kingdom

Les échanges avec des collègues. (Discussions with colleagues)

Psychiatrist, Senegal

#### Theme 3: Enhancement of Clinical Processes and Outcomes

This theme was developed from a review of text where codes related to clinical activities and patient experience (eg, comfort, attendance, and anonymity) were applied. The following 2 subthemes were included:

#### Improves Clinical and Therapeutic Activities

Some clinicians reported that various clinical processes were improved with telehealth. For example, a psychologist in the United States described “easier access to internet search while in therapy to support therapy.” This also included progress tracking, maintaining the alliance, psychoeducation, and follow-ups. Other clinicians described the benefits of having access to patients’ homes and family life along with greater family or caregiver involvement:

Useful to see in client’s homes and the conditions in which they live.

Social worker, Canada

Consultation avec des parents séparés qui ne seraient pas venus ensemble en presence. (Consultations with separated parents who would have never attended together in person.)

Psychiatrist, France

Others reported treatment benefits for specific diagnoses. For example:

Interventions with patients who have Obsessive Compulsive Disorder are far more effective, because patient is in their own home and can practice behavioural exposure during the session.

Psychologist, Canada

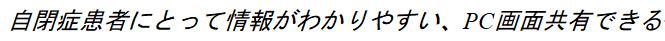
 (Easy for autistic patients to understand information and share PC screen.)

Psychiatrist, Japan

#### Improves Patient Comfort, Attendance, and Engagement

Several clinicians from nearly all WHO regions indicated that telehealth improved patient attendance. For example, a psychologist in New Zealand noted, “better adherence and fewer did not attend!” Some differences among WHO regions were noted. Many of the responses around patient comfort came from providers in the Americas-North region. Clinicians discussed patient comfort in various ways:

Some patients prefer it, and are more comfortable/relaxed their own space than they would be in a therapy room—so they engage better.

Psychologist, Australia

Some clients have felt more comfortable disclosing vulnerable material.

Psychologist, the United States

Nearly all of the responses related to the benefit of patient privacy and anonymity were provided by clinicians in the South-East Asia and Western Pacific-Asia regions and described as an advantage of telehealth:

Some subjects did express that they are happy to have online telehealth services as they do not need to feel noticed by their peers and general public visiting a mental health professional.

Counselor, India

People can be anonymous for those who wants to keep their identity anonymous.

Psychologist, Philippines

#### Other Notable Codes

##### Disadvantages or No Additional Benefits

Although the framing of the short-answer questions was focused on the benefits of telehealth, several providers (58/496, 11.9% responses) indicated that there were no additional benefits aside from remote consultations or exclusively reported disadvantages of telehealth. The vast majority of responses to which this code was applied came from clinicians in Europe, Americas-South, and Western Pacific-Asia. Most of these responses were brief (eg, “no” and “none”), while only a few responses were more descriptive:

No. The Covid experience has confirmed to me that I don’t think it’s a useful longterm alternative to in person therapy.

Psychologist, New Zealand

The only benefit is that the service can be provided despite COVID, but I believe we lose out on many of the small, but important nuances of in-person therapy when limited to on-line intervention.

Psychologist, Canada

##### Positive Disruption of Mental Health Profession

A few clinicians, particularly providers in the Americas-North region, described a systemic level benefit, suggesting that the telehealth shift had challenged and advanced the mental health field and profession by altering service delivery, lifting licensing restrictions, and “kickstarting” new areas:

*Offre aux patients un modèle de souplesse et de flexibilité; permet d’approfondir les enjeux autour des limites et des frontière.(Offers patients a model of flexibility; allows for the exploration of issues around limits and boundaries.)* [Psychologist, Canada]

In South Africa, remote consulting was not permitted by the medical regulatory body. We hope that this will change in future, so that psychiatric services can be made accessible to underserved areas where there are no psychiatrists. This would benefit particularly poor and disadvantaged areas. This can also be a way of upskilling primary care providers in these areas.

Psychiatrist, South Africa

### Comparing Quantitative and Qualitative Results

A joint display of the 2 forms of data offered a complimentary perspective on the concerns and benefits of telehealth according to clinicians [32]. Table 6 presents these contradictory findings as thematic intersections.

**Table 6:** Integrating qualitative and quantitative findings from mental health professionals to generate complementary insights.

	Benefits of telehealth (qualitative)	Concerns with telehealth (quantitative)	Provider- and system-level considerations when evaluating the trade-off
Thematic intersection 1	Improves clinical and therapeutic activities (eg, follow-ups, greater family involvement, more insight into patients’ home and family life, easier use of online tools to support therapy, and diagnosis-specific treatment benefits; 52/496, 10.5% responses)	Clinical effectiveness compared to in-person (506/806, 62.8%), especially, Loss of clinical information (396/500, 79.2%) Building and maintaining the therapeutic alliance (351/500, 70.2%) Showing empathy (237/500, 47.4%) Implementing therapeutic interventions (208/500, 41.6%)	Patient characteristics including disorder or diagnosis, age group, and treatment modality (individual vs group or family or couple) Provider characteristics including telehealth training and experience; telehealth strategies tailored to meet discipline-specific clinical needs Evaluating the loss versus the gain of clinical information Would telehealth enhance or impede therapeutic interventions? Role of flexible blended models that incorporate telehealth and in-person
Thematic intersection 2	Expands reach of mental health services (64/496, 12.9% responses) Timely access to emergency services (6/496, 1.2% responses)	Assessing high-risk patients and managing emergencies remotely (460/806, 57.1%) Remote assessment of patients (358/806, 44.4%)	Patient characteristics including reliable access to technology and internet; technological literacy Provider characteristics including telehealth training and experience Regional characteristics: is this an area or population with no access to mental health services? Are there discipline-specific clinical needs such as infrastructure for physical examination? Opportunity for telehealth models to work closely with providers from remote regions for in-person care and capacity building?
Thematic intersection 3	Improves patient attendance (40/496, 8.1% responses) Improves patient comfort (eg, more at ease in their own homes and more disclosures; 25/496, 5% responses)	Decreased patient engagement compared to in-person (340/806, 42.2%) Lack of private space for patients during telehealth sessions (387/806, 48%)	Patient characteristics including disorder or diagnosis, age group, treatment modality, preferences, and living circumstances Will the lack of privacy in the home environment be an issue? Regional characteristics: could the privacy or anonymity of telehealth help reduce stigma challenges about mental illness and seeking care?

## Discussion

### Overview

This study used quantitative and qualitative methodologies to comprehensively examine and expand upon our understanding of the perspectives, benefits, and concerns related to telehealth service provision among a large, international, and multilingual sample of mental health professionals during the COVID-19 pandemic (November-December 2020). Participants were mainly psychologists and psychiatrists working in mental health settings and providing mental health services such as diagnostic assessment or psychotherapy via telehealth. Most psychiatrists had also provided psychopharmacotherapy services. Half of participating clinicians had less than a year of telehealth experience, suggesting that they likely adopted telehealth because of the pandemic. A little over half of the participants had received some form of telehealth training. Participants were mostly using videoconferencing and/or telephone. This study offers a global snapshot of telehealth implementation from the perspective of clinicians with both previous telehealth experience and new adopters. Findings suggest several important conclusions that can inform recommendations for training and practice for telehealth in mental health care.

### Clinicians Are Primarily Concerned About Clinical Effectiveness and Technological Challenges

Consistent with previous research, clinical effectiveness relative to in-person services was a top concern among clinicians [2,18,38]. However, this study further highlights that clinicians’ primary concerns about clinical effectiveness were regarding the loss of clinical information (eg, nonverbal behavior) and challenges in building and maintaining a therapeutic alliance. Assessment of high-risk patients and managing emergencies remotely was another common clinical concern. These findings support previous research that indicates that major limitations of telehealth practice are reduced availability of clinical data to support assessment or diagnosis [26] and greater difficulty picking up on nonverbal cues [1]. Diminished sense of connection and therapeutic alliance [18,19] has also been raised as a notable challenge associated with telehealth practice, despite this not being fully reflected among findings in the telehealth efficacy literature [39].

With respect to technological concerns, technical issues (eg, audio or video quality and stability of internet connection) were the top concerns followed by patient-related factors such as patients’ access to equipment and familiarity with connecting. The COVID-19 pandemic compelled clinicians to adapt to technology and the technical issues inherent to telehealth. Current best-practice guidelines suggest that proactively establishing backup means of communication with clients can help mitigate these challenges [10]. However, the pandemic has also exposed disparities in access to mental health care among vulnerable populations across low-income and middle-income countries (LMICs) as well as within high-income countries [6]. Hence, client-specific challenges such as technology access, reliability, and competency remain significant barriers to telehealth.

Overall, these top concerns are broadly consistent with the literature [1,18]. However, the current findings build on previous research by examining how demographic and professional characteristics influence the endorsement of common concerns and perceptions, offering further context and insight. For instance, a lack of telehealth training was associated with increased endorsement of common concerns such as clinical effectiveness of telehealth relative to in-person services and remote assessment of patients. Likewise, those with minimal experience using telehealth (ie, less than 1 year) were more likely to report technical difficulties and clinical effectiveness of telehealth relative to in-person services as a concern. These findings support the literature, suggesting that experience and training are related to provider perceptions of telehealth [2,13,40].

### Telehealth Offers Valuable Benefits for Patients and Clinicians That Are Consistent Across Global Contexts

Qualitative data revealed numerous advantages to virtual mental health care. These benefits fell into 3 major areas: accessibility and reach of mental health services, efficiency and flexibility for clinicians, and enhancement of clinical process and outcomes. These main benefits broadly align with existing studies conducted across LMICs and higher-income countries [1,17,18,21,26,41]. However, this international sample extends current research by highlighting the significant advantages of telehealth cited by clinicians from nearly all WHO regions. Specifically, remote mental health care is convenient and flexible for patients because it reduces burdens and barriers to accessing care. Clinicians’ responses illustrated the various economical, geographical, and logistical barriers that clients regularly encounter when accessing in-person mental health care. Moreover, telehealth improved patient access by enabling clinicians to see more patients and extend specialized services to a broader population. Thus, continued implementation of telehealth services alongside in-person care can help ensure equitable mental health access.

Based on participants’ perceptions, telehealth also offers convenience and flexibility for mental health professionals by giving them greater control over their time and clinical outreach. Previous studies have spoken about the personal benefits of telehealth for mental health professionals including more efficient use of time [18] and improved work-life balance [22]. This study further illustrates how telehealth can support clinician efficiency (eg, no commuting), well-being and quality of life (eg, working in a relaxing home environment), and professional development. As such, telehealth offers valuable benefits for mental health professionals, particularly for clinicians at life stages where flexibility and work-life balance are important. This is likewise relevant to occupational stress and burnout, as the COVID-19 pandemic underscored the need to safeguard the capacity of the global mental health workforce [31]. Health care systems and clinicians should leverage the above benefits to promote provider efficiency and health, such as policies supporting hybrid work models, while maintaining quality care.

Increased safety from COVID-19 and improved patient attendance were among the most prominently reported benefits among participants. The latter is consistent with the literature [21,22]. The reduction of barriers to access likely facilitates client attendance. Altogether, qualitative findings underscore why telehealth has become a routine practice for many clinicians in a postpandemic era.

### Psychologists and Psychiatrists Engage Differently With Telehealth

Our quantitative data also revealed informative data regarding professional differences between psychologists’ and psychiatrists’ use (Table 2) and perceptions of telehealth. Findings indicated that psychologists spent significantly more hours delivering clinical services via videoconferencing and telephone than psychiatrists. Conversely, there were no significant differences in the number of patients seen between the 2 disciplines across telehealth modalities. With respect to endorsement of telehealth concerns, psychiatrists were more likely to report challenges with remote assessment of patients, assessing high-risk patients and managing emergencies, showing empathy, and licensing or credentialing requirements compared to psychologists. Conversely, psychologists were more likely to be concerned about a lack of private space for patients during telehealth sessions compared to psychiatrists.

These findings likely reflect differences in the clinical services each discipline provides. Psychiatrists’ concerns emphasize their need for additional infrastructural support for adapting in-person interventions like physical examinations or blood pressure monitoring [26]. Moreover, participating psychiatrists provided other clinical activities such as medication management or general medical services that are briefer in nature compared to assessment or psychotherapy services. Likewise, psychologists, who spend more direct time with patients (eg, providing psychotherapy), may prioritize ensuring that patients have a private space conducive to treatment. Altogether, these findings stress the need for telehealth strategies that are tailored to the needs of psychologists and psychiatrists. This could entail specialized telehealth training, telehealth models that integrate in-person and remote care, and improved regulatory frameworks to support remote psychiatric care. Likewise, platforms that optimize usability to accommodate different clinical needs and policies that focus on improving the standards and accessibility of telehealth may be beneficial.

### Telehealth Service Delivery Involves Balancing Trade-Offs

An integration of quantitative and qualitative findings (Table 6) illustrated that there was much overlap among concerns and benefits of telehealth. For instance, some clinicians noted that telehealth provided valuable clinical insights into patients’ home and family environments, while others endorsed concerns about a loss of clinical information, such as nonverbal cues. Likewise, providers observed improved attendance, engagement, and continuity of care. However, clinicians also worried about the therapeutic alliance and patient engagement. This overlap in perspectives has been reported in the literature [22,25,40]. Data from the international sample of mental health professionals presented here offer a nuanced perspective: there are inherent trade-offs between telehealth’s benefits and disadvantages.

It is important for health care systems and stakeholders to be aware of the potential advantages and disadvantages of telehealth practice when planning mental health service. Flexible telehealth models can harness advantages while addressing concerns about clinical effectiveness and technology through hybrid or capacity-building initiatives that integrate available in-person support within patient communities (eg, medical personnel for physical examinations, psychometrist for cognitive assessments, and staff for crisis management).

At the provider level, clinicians must evaluate telehealth’s trade-offs to determine its suitability for each patient. Will telehealth enhance or limit therapeutic outcomes? The available literature recommends evaluating provider competencies, patient needs and preferences, and organizational resources when determining the appropriateness of telehealth among other considerations [42]. For instance, this study found that perceived effectiveness of telehealth was higher for individual assessment and treatment as well as adult-aged patients. Likewise, service modality, particularly family or group therapy, or initial assessment may impact rapport building [39]. Research also suggests that having the initial patient interaction be in-person can help establish rapport prior to telehealth services [25,43]. Finally, adapting clinician communication style to include greater use of vocal tone and facial expression can help convey empathy [43].

### Differences Among WHO Regions

Quantitative findings identified that participants from South-East Asia and Western Pacific-Asia were more likely to endorse concerns related to remote assessment and patient privacy, security, and confidentiality when compared to participants in the Americas-North. Conversely, participants from the Americas-South and Western Pacific-Asia were less likely to report concerns about patient privacy, security, and confidentiality, and technical difficulties, respectively. Qualitative findings revealed that clinicians in Europe, Americas-South, and Western-Pacific Asia reported disadvantages to telehealth (eg, inferiority to in-person care and difficulty obtaining payment for services) or simply indicated no additional benefits aside from remote contact. Reasons for variations in participant impressions across WHO regions are likely multifaceted; however, a few factors can be considered. First, the pandemic necessitated an immediate, global shift to telehealth with almost complete virtualization of many services at the time of data collection [7], meaning that many clinicians were faced with adopting unfamiliar telehealth platforms. As such, WHO regions varied in readiness for such changes in practice and training. For instance, in a survey study with medical students, trainee and early career psychiatrists from South-East Asia and Western-Pacific Asia regions conducted in early 2021 found limited opportunities for training and knowledge of telehealth among young professionals [41]. Some regions also faced key barriers to uptake including reservations about telehealth among clinicians, inadequate communication infrastructure (eg, in some parts of Latin America), and lack of regulatory frameworks (eg, professional licensing) in some parts of Europe [44-46].

Qualitative data also found that privacy and anonymity as benefits of telehealth were primarily noted by participants in the South-East Asia and Western Pacific-Asia regions—advantages that have been less addressed in the literature. Challenges related to stigma around mental illness in these regions may explain this heightened benefit among its mental health professionals [47]. A few participants, particularly those in the Americas-North, believed that the abrupt shift to telehealth had advanced the field of mental health. Quantitative data identified that clinicians in the Americas-North were more likely to be concerned about a lack of private space for patients during telehealth sessions compared to clinicians from Western Pacific-Asia. These findings may reflect how certain regions, such as North America—where much telehealth research has been conducted [23,26], had more familiarity with the implementation of virtual models.

Countries and regions must now reflect on the future direction of telehealth. This study suggests that to capitalize on telehealth’s broad advantages, investing in telehealth training and education and the dissemination of resources for mental health professionals are crucial. Countries should also address disparities in access to digital technology, known as the “digital divide,” that affects LMICs and high-income countries. Regional telehealth models could promote digital literacy among specific patient groups, assess the use of tools that require less bandwidth (eg, telephone and instant messaging), or offer stability and privacy for telehealth services through local health centers or schools.

### Limitations

This study has some limitations. Study findings were based on provider perspectives rather than objective measures of quality of mental health care or ethical practices. It will be important to further examine the patient perspective and assess the quality of services delivered. Given that data were gathered during the early stages of the pandemic, clinicians’ perceptions and responses were likely impacted by their evolving contexts at the time. Furthermore, the quality of telehealth training indicated by clinicians was unknown. Since participant perceptions were based on multiple telehealth modalities (synchronous and asynchronous) and clinical contexts, direct comparisons (eg, telephone vs videoconferencing) were not possible. As such, present findings remain general and based on clinicians’ own combination of telehealth service modalities provided in a public health emergency. Although this study had representation from all WHO regions and country income levels, most participating clinicians were from high- and upper-middle–income countries. Finally, given the cross-language qualitative data, it is possible that translation decisions and coders being outside of the sociocultural environments of many participant clinicians (coders were based in North America) impacted interpretation. Although the number of short-answer responses in Japanese or Chinese was small (roughly 44/496, 9%), the DeepL translations were not reviewed by fluent speakers, which may limit interpretive accuracy. Future research should explore implementation and patient outcomes within specific telehealth modalities, clinical settings, and geographical contexts, particularly its potential in LMICs.

### Conclusions

The results of this study suggest that telehealth for mental health services presents notable clinical and technological challenges that are common across global contexts. Certain competencies such as telehealth training and experience may help mitigate these concerns. Moreover, given that psychologists and psychiatrists seem to engage with telehealth differently, specialized training and tailored approaches may also help implementation. Nonetheless, results also suggest that telehealth offers valuable benefits for patients and providers. Blended models that integrate virtual and in-person care or capacity-building platforms for underserved areas are worth exploring. In a postpandemic era with more deliberate implementation of telehealth services and a new generation of digitally trained clinicians, we can balance its benefits and limitations to optimize mental health care and clinical outcomes globally.

## Appendix

Multimedia Appendix 1 GRAMMS checklist.

Multimedia Appendix 2 Survey questions.

## Abbreviations

GCPN: Global Clinical Practice Network
GRAMMS: Good Reporting of a Mixed Methods Study
ICD-11: International Classification of Diseases, 11th Revision
LMIC: low-income and middle-income country
WHO: World Health Organization

## Data Availability

The datasets generated or analyzed during this study are not publicly available due to the nature of our original ethics approval. Anonymized, group-level data are available from the corresponding author on reasonable request.
