# Supplementary material for: Mental Health Professionals’ Perceptions of Benefits and Disadvantages of Telehealth: International Mixed Methods Study
Source: J Med Internet Res. 2026 Mar 4;28:e75905. doi: 10.2196/75905 (PMC13000382; doi:10.2196/75905)
Supplement: Multimedia Appendix 1 [file jmir_v28i1e75905_app1.docx]

| **Guideline** | **Section: page in the manuscript** |
| --- | --- |
| 1. Describe the justification for using a mixed methods approach to the research question | Introduction: p 7  The present mixed-methods study used distinct but complementary qualitative and quantitative research questions to obtain more comprehensive information on the experiences, concerns and benefits of telehealth from the perspective of international mental health providers during the COVID-19 pandemic. |
| 1. Describe the design in terms of the purpose, priority and sequence of methods | Methods: p 8  A convergent mixed methods design was used in which quantitative and qualitative data were collected in parallel (i.e., in the same survey), analysed separately with equal priority, and then merged. |
| 1. Describe each method in terms of sampling, data collection and analysis | Methods: p. 8  Quantitative: close-ended survey items with mental health professionals  Analyses: p 10.  Qualitative: open-ended survey question with mental health professionals  Analyses: P 11. |
| 1. Describe where integration has occurred, how it has occurred and who has participated in it | Methods: p 13. |
| 1. Describe any limitation of one method associated with the present of the other method | Limitations: p 35. |
| 1. Describe any insights gained from mixing or integrating methods | Table 6 illustrating integration of findings  Integrating methods show telehealth trade-offs p 26. |

*O'Cathain A, Murphy E, Nicholl J. The quality of mixed methods studies in health services research. J Health Serv Res Policy. 2008;13(2):92-98.*
