# Supplementary material for: Mental Health Professionals’ Perceptions of Benefits and Disadvantages of Telehealth: International Mixed Methods Study
Source: J Med Internet Res. 2026 Mar 4;28:e75905. doi: 10.2196/75905 (PMC13000382; doi:10.2196/75905)
Supplement: Multimedia Appendix 2 [file jmir_v28i1e75905_app2.docx]

Below are select telehealth items drawn from the online survey administered between November 11 and December 18, 2020. The broader survey examined not only telehealth service delivery but also changes in work circumstances, occupational stressors and well-being, and clinicians’ expectations and recommendations for future practice. The survey also included branching logic for some questions.

**For approximately how many years have you been providing telehealth services (i.e, assessment, treatment and monitoring of patients)?** Do not include routine contact such as scheduling appointments and refilling medications.

▼ Less than 1 year, 1,2,3... 20

**During the PAST TWO WEEKS, which telehealth tools have you used to deliver services to patients (i.e., assessment, treatment or monitoring)?** Do not include routine contact such as scheduling appointments and refilling medications. (Select all that apply.)

- Telephone
- Video conferencing (e.g., Skype, Zoom, GoToMeeting)
- Chat or instant messaging (e.g., WhatsApp, WeChat, text messages)
- E-mail
- Other (please specify:) ________________________________________________

*If “telephone” was selected:*

**How many patients did you assess, treat or monitor in the PAST TWO WEEKS via telephone?** Please count each patient only once, even if you saw them multiple times during this period.

▼ 1 ... More than 100

**During the PAST TWO WEEKS, how many hours did you spend assessing, treating or monitoring patients via telephone?**

▼ Less than 1 hour ... More than 100

*f*

*If “videoconferencing” was selected:*

**How many patients did you assess, treat or monitor in the PAST TWO WEEKS via videoconferencing (e.g., Skype, Zoom, GoToMeeting)?** Please count each patient only once, even if you saw them multiple times during this period.

▼ 1 ... More than 100

**During the PAST TWO WEEKS, how many hours did you spend assessing, treating or monitoring patients via videoconferencing (e.g., Skype, Zoom, GoToMeeting)?**

▼ Less than 1 hour ... More than 100

*If “chat or instant messaging” was selected:*

**How many patients did you assess, treat or monitor in the PAST TWO WEEKS via chat or instant messaging (e.g., WhatsApp, WeChat, text messages)?** Please count each patient only once, even if you saw them multiple times during this period.

▼ 1 ... More than 100

**During the PAST TWO WEEKS, how many hours did you spend assessing, treating or monitoring patients via chat or instant messaging (e.g., WhatsApp, WeChat, text messages)?**

▼ Less than 1 hour ... More than 100

*If “email” was selected:*
**How many patients did you assess, treat or monitor in the PAST TWO WEEKS via e-mail?** Please count each patient only once, even if you saw them multiple times during this period.

▼ 1 ... More than 100

**During the PAST TWO WEEKS, how many hours did you spend assessing, treating or monitoring patients via e-mail?**

▼ Less than 1 hour ... More than 100

**Have you received specific training on the following aspects of telehealth service delivery?** (Select all that apply.)

- **Technological aspects** (e.g., internet speed and connectivity, choice of platform, platform security)
- **Ethical and legal aspects** (e.g., consent for telehealth, privacy, licensing)
- **Clinical aspects** (e.g., adapting treatment protocols, remote assessment)
- ⊗**None**

**Have you been concerned with any of the following clinical elements of providing telehealth services?** (Select all that apply.)

- Clinical effectiveness relative to in-person services (e.g., building and maintaining a therapeutic alliance, showing empathy, setting common goals and collaborating with patients, implementing therapeutic interventions
- Remote assessment of patients (e.g., diagnostic assessments, self-report measures, blood pressure monitoring, lack of opportunity for physical examinations, medication blood level monitoring)
- Assessment of high-risk patients (e.g., suicidal or psychotic) and managing emergencies (e.g., domestic violence) remotely
- Lack of supporting research or awareness of relevant research on telehealth services
- Decrease in patient engagement compared to in-person services
- Lack of private space for patients during telehealth sessions
- Other (please specify:) ________________________________________________
- ⊗None of the above

*If “Clinical effectiveness relative to in-person services…” was selected:*

**Which of the following aspects of the clinical effectiveness of providing telehealth services have you been concerned with?**(Select all that apply.)

- Building and maintaining a therapeutic alliance
- Showing empathy
- Setting common goals and collaborating with patients
- Loss of clinical information (e.g., interpreting non-verbal behavior of patient)
- Implementing therapeutic interventions (e.g., homework, exposures)
- Other (please specify:) ________________________________________________

**Have you been concerned with any of the following technical elements of providing telehealth services?** (Select all that apply.)

- Your access to equipment (e.g., devices, computers, video cameras, internet access)
- Patients’ access to equipment (e.g., devices, computers, video cameras, internet access)
- Technical issues (e.g., audio/video quality, stability of internet connection)
- Ability to access and update patient records remotely
- Inadequate technical (IT) and administrative support
- Your familiarity with managing teleconference scheduling and session controls (e.g. muting, video, audio)
- Patient familiarity with connecting to teleconference sessions and managing session controls
- Dissatisfaction with choice of telehealth platform or platform security
- Other (please specify:) ________________________________________________
- ⊗None of the above

|  |
| --- |

**Have you been concerned with any of the following ethical, legal and professional elements of providing telehealth services?** (Select all that apply.)

- Patient consent, privacy, security, and confidentiality
- Lack of direction from my professional association
- Licensure/credentialing requirements
- Legal and regulatory issues (e.g., liability, malpractice, providing services in other jurisdictions)
- Other (please specify:) ________________________________________________
- ⊗None of the above

**Have you been concerned with any of the following administrative elements of providing telehealth services?** (Select all that apply.)

- Disruption of routine and workflow (e.g., additional workload caused by scheduling, instructing patients on use of telehealth services)
- Lack of sufficient time to learn how to use technology effectively
- Billing and reimbursement for services you provide
- Cost of subscription (if not provided by institution)
- Other (please specify:) ________________________________________________
- ⊗None of the above

**In addition to making it possible to consult with patients remotely, have there been any benefits of using telehealth services?** (Please describe briefly.)

________________________________________________________________

________________________________________________________________

________________________________________________________________

________________________________________________________________

________________________________________________________________

**Overall, how effective do you think you have been in providing clinical services via telehealth in the PAST TWO WEEKS?**

- Very ineffective
- Somewhat ineffective
- Somewhat effective
- Very effective

**Relative to in-person services, how effective have telehealth services been for the following patient age groups?**

|  | I have not provided telehealth services to this group | About the same | Not as effective as in-person services | More effective than in-person services |
| --- | --- | --- | --- | --- |
| **Children (0-12 years)** |  |  |  |  |
| **Adolescents (13-18 years)** |  |  |  |  |
| **Adults (19-64 years)** |  |  |  |  |
| **Older adults (65+ years)** |  |  |  |  |

**Relative to in-person services, how effective have telehealth services been for the following treatment modalities?**

|  | I have not provided telehealth services to this group | About the same | Not as effective as in-person services | More effective than in-person services |
| --- | --- | --- | --- | --- |
| **Individual assessment and treatment** |  |  |  |  |
| **Couples** |  |  |  |  |
| **Families** |  |  |  |  |
| **Groups** |  |  |  |  |
